# Prediction of malignant intraductal papillary mucinous neoplasm: A nomogram based on clinical information and radiological outcomes

**DOI:** 10.1002/cam4.6326

**Published:** 2023-07-11

**Authors:** Xiaorui Huang, Tong Guo, Zhiwei Zhang, Ming Cai, Xinyi Guo, Jingzhao Zhang, Yahong Yu

**Affiliations:** ^1^ Department of Biliopancreatic Surgery Tongji Hospital of Tongji Medical College of Huazhong University of Science and Technology Wuhan China

**Keywords:** intraductal papillary mucinous neoplasm, malignancy prediction, nomogram, prognostic nutritional index

## Abstract

**Objective:**

Clinical practitioners face a significant challenge in maintaining a healthy balance between overtreatment and missed diagnosis in the management of intraductal papillary mucinous neoplasm (IPMN). The current study aimed to identify significant risk factors of malignant IPMN from a series of clinical and radiological parameters that are widely available and noninvasive and develop a method to individually predict the risk of malignant IPMN to improve its management.

**Methods:**

We retrospectively investigated 168 patients who were pathologically diagnosed with IPMN after individualized pancreatic resection between June, 2012 and December, 2020. Independent predictors determined using both univariate and multivariate analyses to construct a predictive model. The discriminatory power of the nomogram was assessed using the area under the receiver operating characteristic curve (AUC). Decision curve analysis was performed to demonstrate the clinical usefulness of the nomogram. Internal cross validation was performed to assess the validity of the predictive model.

**Results:**

In the multivariate analysis, five significant independent risk factors were identified: increased serum CA19‐9 level, low prognostic nutritional index (PNI), cyst size, enhancing mural nodule, and main pancreatic duct diameter. The nomogram based on the parameters mentioned above had outstanding performance in distinguishing malignancy, with an AUC of 0.907 (95% confidence interval: 0.859–0.956, *p* < 0.05), which remained 0.875 after internal cross‐validation, and showed good clinical usefulness.

**Conclusion:**

A novel nomogram for predicting malignant IPMN first introducing PNI was developed, which may aid in improving IPMN management. Nevertheless, external validation is required to confirm its efficacy.

## Abstract 2

With the hope of improving the management of intraductal papillary mucinous neoplasm (IPMN) of the pancreas, we developed a new malignancy‐predicted nomogram for IPMN, based on five verified independent predictors of malignant IPMN in our study. We employed Prognostic nutrition index (PNI) to constructed the nomogram for the first time and stratified the diameter of main pancreatic duct and the size of Mural Nodules which were helpful to achieve a more precise risk for malignancy. The nomogram shows excellent capability in predicting malignant IPMN and has a promising application potential.

## 1 INTRODUCTION

Owing to the rapid development and widespread application of cross‐sectional imaging technology and perfection of disease identification and classification, the radiological detection of intraductal papillary mucinous neoplasms (IPMNs) has greatly increased. IPMNs are thought to make up the majority of asymptomatic pancreatic cystic neoplasms (PCNs)^1^, ^2^ and are one of the most important precursors of pancreatic carcinoma, which has an underlying tendency to progress from low‐grade dysplasia (LGD) to high‐grade dysplasia (HGD) and even invasive carcinoma.^3^, ^4^ Compared with another pancreatic precursor, pancreatic intraepithelial neoplasia, which is detected only through histopathological examination,^5^ IPMN as a cystic lesion can be detected using cross‐sectional imaging. Although the concrete mechanisms driving progression remain unclear,^6^ the indolent nature of IPMN makes it possible to provide early intervention for patients with IPMN. Considering the nature of IPMN progression, the ideal strategy in the management of IPMN might be timely surgical intervention for high‐risk IPMN (HGD and invasive carcinoma) to prevent tumor progression and improve patient prognosis and conservative surveillance for asymptomatic low‐risk IPMN (LGD) to avoid unnecessary surgical procedures and reduce damage to the patients.^7^, ^8^ Nevertheless, the main issues that plague clinicians are the preoperative assessment of the malignant risk of IPMN and determination of the timing of surgery. Therefore, clinicians face significant challenges in maintaining a balance between avoiding excessive surgical procedure and missing malignancies during conservative surveillance in the management of IPMN.

In the last decade, several consensuses and guidelines, including the 2015 American Gastroenterological Association (AGA) guidelines,^9^ 2017 International Association of Pancreatology (IAP) guidelines^7^ and 2018 European Study Group on Cystic Tumors of the Pancreas (ESG) guidelines,^10^ have proposed constructive recommendations for resection indications or surveillance strategies. At the same time, discordance exists among these current guidelines, and none of the guidelines can accurately identify malignant IPMN, bringing about a series of controversies regarding the management of IPMN. Hence, the proportion of patients undergoing unnecessary pancreatectomy for low‐risk IPMN remains considerable. Adequate assessment is significantly necessary before performing aggressive surgery for patients with IPMN, considering the 2%–4% risk of mortality and 20%–25% risk of major morbidity after pancreatic resection.^11^

To improve the management of IPMN, several nomograms incorporating various predictors have been developed to assess the malignancy risk of IPMN,^12^, ^13^, ^14^, ^15^, ^16^, ^17^, ^18^ which appear to be beneficial. However, some of them were only applicable to the simplex subtype, either main duct‐IPMN (MD‐IPMN) or branch duct‐IPMN (BD‐IPMN).^15^, ^17^, ^18^ In some cases, it is difficult to confirm the exact subtype of IPMN in the preoperative images. Besides, Waters et al. reported that in the diagnosis of the subtype of IPMN, the correlation between the radiologic criteria and pathologic evaluations was approximately 70%.^19^ Additionally, the majority of the nomograms included mural nodules, which were mainly detected using endoscopic ultrasound (EUS). However, as an invasive examination, EUS is not available at some institutions, despite the fact that it is thought to be the most reliable way to detect the presence of a mural nodule and measure its size.^20^, ^21^ In the current study, we proposed the construction of a nomogram that can be applied to all subtypes of IPMN to predict malignancy based on preoperative clinical data, including laboratory examinations and radiological features, that are noninvasive and generally available.

## 2 MATERIALS AND METHODS

**FIGURE 1:**
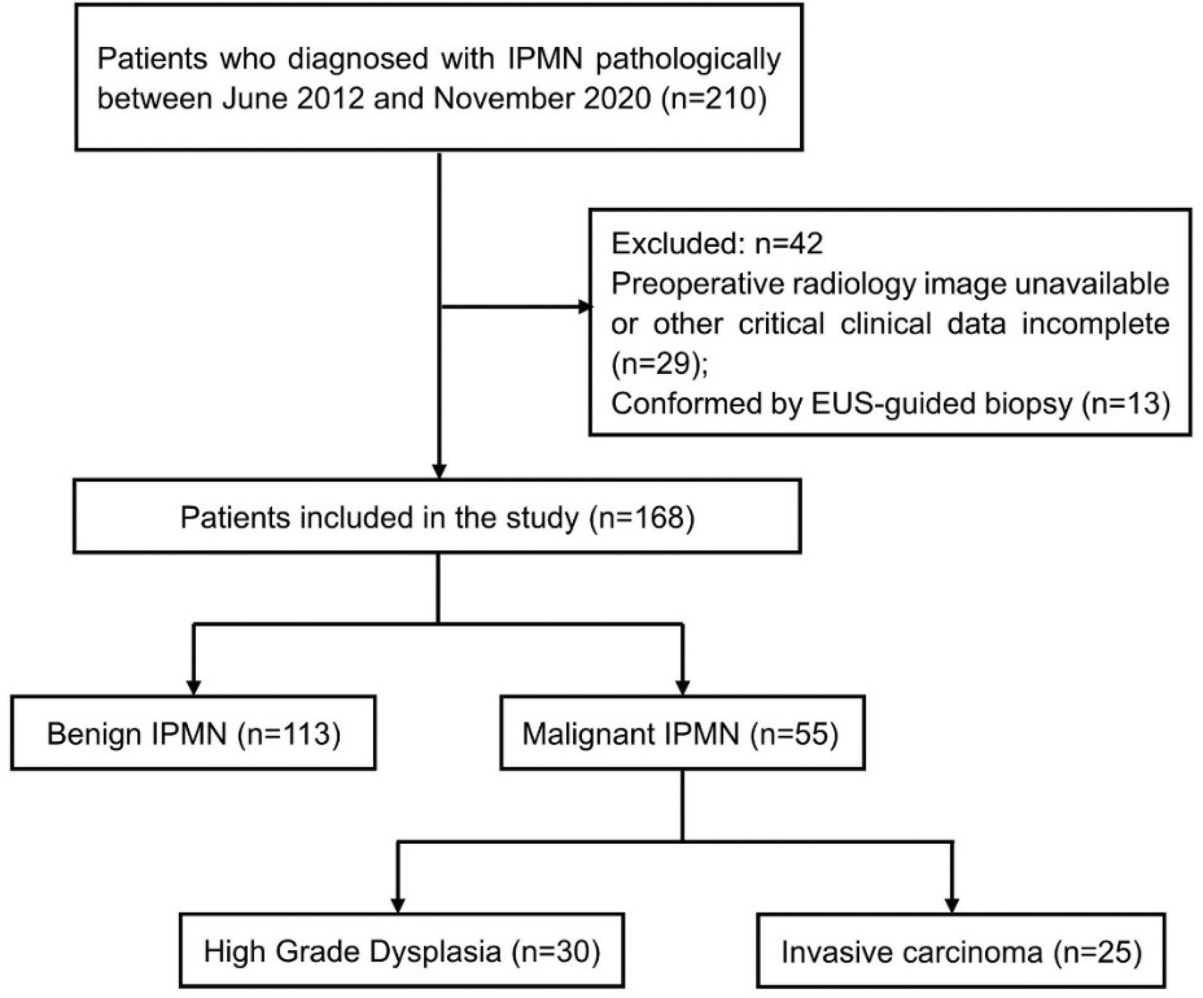
Flowchart of patient selection.

The current study was a retrospective study investigating 210 patients who were pathologically diagnosed with IPMN at the Department of Biliopancreatic Surgery, Tongji Hospital, Tongji Medical College, Huazhong University of Science and Technology between June, 2012 and December, 2020. The inclusion criteria were as follows^1^: pathological evidence obtained from resected specimens after surgery,^2^ related preoperative laboratory results available, and^3^ preoperative cross‐sectional contrast‐enhanced computed tomography (CT) or magnetic resonance imaging (MRI) images that were clearly visible. The exclusion criteria were as follows^1^: diagnosis through EUS‐guided biopsy, without further confirmation of the resected specimens after surgery,^2^ incomplete preoperative critical laboratory tests (CA19‐9 and CEA),^3^ unavailable preoperative radiology imaging or failure to identify the critical features from the low‐quality images, and^4^ concomitant pancreatic duct adenocarcinoma. Finally, 168 patients were included in the study (Figure 1).

Data on demographics, clinical manifestations (such as jaundice, history of acute pancreatitis, and diabetes), laboratory tests (neutrophils, lymphocytes, platelets, albumin, glucose, total bilirubin, serum CA19‐9, and CEA), imaging features, surgical protocols, and pathological outcomes were gathered from our electronic medical record system and picture archiving communication system. Body mass index (BMI) was calculated as weight in kilograms (kg) divided by height in meters squared (m^2^). The neutrophil‐to‐lymphocyte ratio (NLR) was defined as the absolute neutrophil count (10^9^/L) divided by the absolute lymphocyte count (10^9^/L), while the platelet‐to‐lymphocyte ratio (PLR) was defined as the absolute platelet count (10^9^/L) divided by the absolute lymphocyte count (10^9^/L).^22^ The prognostic nutritional index (PNI) was calculated as follows: albumin (g/L) + 5 × absolute lymphocyte count (10^9^/L).^23^

Blinded to the postoperative pathological diagnosis, two skilled radiologists, with 10 and 15 years of experience in biliary and pancreatic imaging, respectively, reviewed the preoperative images of patients with IPMN and identified target characteristics. When they disagreed on certain aspects, a third senior radiologist assessed the case and rendered the final judgment.

The following radiological characteristics were evaluated according to the 2017 IAP and 2018 ESG guidelines. Based on imaging, IPMN was classified into three types: MD type, BD type, and mixed type (MT). MD‐IPMN is characterized by segmental or diffuse dilation of the main pancreatic duct (MPD) of ≥5 mm, without other causes of obstruction. Pancreatic cysts of ≥5 mm in diameter that communicate with the MPD were identified as BD‐IPMN, with pseudocysts being the differential diagnosis for patients with a prior history of pancreatitis. Patients with MT‐IPMN met the criteria for both MD‐IPMN and BD‐IPMN.^7^ Tumor location was divided into head/uncinate, body/tail, or diffuse. The cyst size was measured at the maximum diameter of the cross‐section. When multiple cysts were present, the maximum diameter of the largest cyst was recorded if they were located in the same site, or each branch cyst would be accessed and recorded separately if these cysts appeared multifocal. Mural nodule was defined as any enhancing solid component protuberance within either the branch cyst or dilated MPD, whose size categorized into 0 (no mural nodule), <5 mm, or ≥5 mm. The maximum MPD diameter was recorded. Other characteristics that might be recognized in the lesions included an abrupt change in the MPD caliber with distal pancreatic atrophy (a ratio between the diameter of the MPD and width of the pancreatic parenchyma at the same location >0.5 suggested parenchymal atrophy),^24^ thickened enhancing cyst wall (defined as a thickening of the wall ≥2 mm),^25^ and lymphadenopathy (defined as enlarged peripancreatic lymph nodes, with a maximum short‐axis diameter > 10 mm).^26^

In all patients, the diagnosis of IPMN was confirmed by a pathologist depending on the histological and immunohistochemical findings of the surgical specimens. According to the 2019 World Health Organization histological classification of IPMN, the pathological diagnoses are classified as IPMN with LGD, IPMN with HGD, and IPMN with invasive carcinoma.^3^ The former is considered a benign disease, whereas the latter two are considered malignant. IPMNs with invasive carcinomas were staged depending on the depth of invasive components, according to the AJCC/TNM classification system (eighth edition).

This study was approved by the Institutional Review Board of the Tongji Hospital of Tongji Medical College, Huazhong University of Science and Technology.

### 2.1 Statistical analysis

All statistical analyses were performed using the R software version 4.2.1. For continuous variables, the Shapiro–Wilk test was first performed, values were presented as the mean ± standard deviation for data that were normally distributed or median and interquartile range for data that conformed to the nonnormal distribution. The Mann–Whitney U‐test was used to test nonnormally distributed continuous variables, whereas *t*‐test was used to test normally distributed continuous variables. The categorical variables were evaluated using *χ* ^2^‐test or the Fisher's exact test (when expected frequencies <5) and described as numbers and percentages (%). Continuous variables were converted to categorical variables (Table 1). Logistic regression analysis was implemented, and variables whose *p*‐values were less than 0.05 in the univariate analysis were included in the multivariate analysis. Finally, the variables that remained significant predictors in the multivariate analysis were used to construct a nomogram to predict malignant IPMN. The 200 times three‐fold internal cross‐validation was performed to assess the validity of the predictive model. Receiver‐operating characteristic (ROC) curves were used to measure the predictive accuracy of the nomogram. Calibration curve and decision curve analyses were performed to evaluate the predictive performance of the model. Based on the true positive, false positive, true negative, and false negative results for malignancy diagnosis, the sensitivity, specificity, positive predictive value, negative predictive value, and accuracy were calculated to assess the diagnostic performance of various cut‐off values of malignancy probability for the nomogram. A two‐sided *p* value <0.05 was used to indicate a statistically significant difference.

**TABLE 1:** Continuous variables converted into categorical variables.

Variates	Classification basis	Threshold	Groups or grades
BMI	Criteria of WHO	—	Underweight: < 18.5 kg/m^2^
		18.5	Normal: 18.5–24.9 kg/m^2^
		25	Overweight:25–29.9 kg/m^2^
		30	Obesity: ≥30 kg/m^2^
MPD	2017 IAP guidelines & 2018 ESG guidelines	—	<5 mm
		5	5–9 mm
		10	≥10 mm
MNs	2017 IAP guidelines & 2018 ESG guidelines	—	<5 mm
		5	≥5 mm
Cyst	2017 IAP guidelines	—	<30 mm
		30	≥30 mm
	2018 ESG guidelines	—	<40 mm
		40	≥40 mm
CA19‐9	2017 IAP guidelines & 2018 ESG guidelines	—	<37 U/L
		37	≥37 U/L
CEA	Wang W et al. 2015^27^	—	<5 ng/mL
		5	≥5 ng/mL
NLR	Li B et al. 2021^28^	—	<2
		2	≥2
PLR	Li B et al. 2021^28^	—	<120
		120	≥120
PNI	ROC curve analysis	—	<45.875
		45.875	≥45.875

Abbreviations: BMI, body mass index; ESG, European study group of cystic neoplasm; IAP, international association of pancreas; MNs, mural nodules; MPD, main pancreatic duct; NLR, neutrophils to lymphocytes ratio; PLR, platelets to lymphocytes ratio; PNI, prognostic nutrition index; ROC, receiver operative characteristic.

## 3 RESULTS

### 3.1 Patient characteristics

The demographic and clinicopathological characteristics of patients diagnosed with IPMN after surgery are presented in Table 2. A series of surgical procedures, including laparoscopic or open pancreatoduodenectomy (PD), duodenum‐preserving pancreatic head resection (DPPDR), distal pancreatectomy (DP), middle pancreatectomy (MP), and enucleation, were performed individually in 168 patients suspected of having IPMN based on preoperative radiological findings. The mean age of these patients comprising 100 men (59.5%) and 68 women (40.5%) was 61 years. In total, 62.5% of the lesions were found to be located in the head or uncinate of the pancreas. In final pathological analysis, benign lesions (IPMN with LGD) were identified in 113 of the 168 patients (67.3%), while the remaining 32.7% of the patients were proved malignant, including 25 cases of IPMN with invasive carcinoma and 30 cases of IPMN with HGD. BD‐IPMN accounted for the largest proportion at 54.3% (91/168), followed by MT‐IPMN at 33.3% (56/168) and MD‐IPMN at 12.4% (21/168), respectively. Moreover, 50.0% (37/74) of the patients with MD/MT‐IPMN and 19.1% (18/94) of the patients with BD‐IPMN suffered from malignant disease. However, no significant differences were observed between the benign and malignant groups in terms of age, sex, or lesion location.

**TABLE 2:** Baseline characteristics of patients with IPMN.

Characteristic	All (*n* = 168)	Benign (*n* = 113)	Malignant (*n* = 55)	*p*‐value
Age (years)	61.0 [52.0;66.0]	60.0 [52.0;65.0]	62.0 [53.5;71.5]	0.085
Sex				0.355
Female	68 (40.5%)	49 (43.4%)	19 (34.5%)	
Male	100 (59.5%)	64 (56.6%)	36 (65.5%)	
Surgery				0.003
DP	49 (29.2%)	36 (31.9%)	13 (23.6%)	
DPPHR	29 (17.3%)	24 (21.2%)	5 (9.09%)	
Enucleation	4 (2.38%)	4 (3.54%)	0 (0.00%)	
MP	3 (1.79%)	3 (2.65%)	0 (0.00%)	
PD	74 (44.0%)	44 (38.9%)	30 (54.5%)	
TP	9 (5.36%)	2 (1.77%)	7 (12.7%)	
Location				0.160
Body/tail	54 (32.1%)	41 (36.3%)	13 (23.6%)	
Diffuse	9 (5.36%)	7 (6.19%)	2 (3.64%)	
Head/uncinate	105 (62.5%)	65 (57.5%)	40 (72.7%)	
Type				<0.001
BD	94 (56.0%)	76 (67.3%)	18 (32.7%)	
MD	18 (10.7%)	7 (6.19%)	11 (20.0%)	
MT	56 (33.3%)	30 (26.5%)	26 (47.3%)	
BMI	22.8 (2.98)	22.5 (2.87)	23.2 (3.16)	0.194
BMI grade				
Underweight	17 (10.1%)	10 (8.85%)	7 (12.7%)	
Normal	95 (56.5%)	70 (61.9%)	25 (45.5%)	
Overweight	50 (29.8%)	30 (26.5%)	20 (36.3%)	
Obesity	6 (3.51%)	3 (2.65%)	3 (5.45%)	
NLR	1.73 [1.27;2.52]	1.75 [1.31;2.38]	1.72 [1.25;2.87]	0.618
NLR≥2				0.764
No	102 (60.7%)	70 (61.9%)	32 (58.2%)	
Yes	66 (39.3%)	43 (38.1%)	23 (41.8%)	
PLR	113 [90.3;138]	115 [93.0;135]	112 [88.1;138]	0.886
PLR≥120				1.000
No	105 (62.5%)	71 (62.8%)	34 (61.8%)	
Yes	63 (37.5%)	42 (37.2%)	21 (38.2%)	
PNI	47.2 [43.7;50.3]	48.5 [46.0;52.0]	42.0 [38.4;45.8]	<0.001
PNI≤45.875				0.002
No	88 (52.4%)	69 (61.1%)	19 (34.5%)	
Yes	80 (47.6%)	44 (38.9%)	36 (65.5%)	
CA19‐9	12.6 [7.00;29.6]	8.87 [5.31;15.3]	31.2 [12.7;43.4]	<0.001
CA19‐9 ≥ 37 U/mL				<0.001
No	144 (85.7%)	105 (92.9%)	39 (70.9%)	
Yes	24 (14.3%)	8 (7.08%)	16 (29.1%)	
CEA	2.20 [1.31;3.95]	1.81 [1.21;3.17]	3.64 [2.02;4.84]	<0.001
CEA**≥**5 ng/mL				0.036
No	146 (86.9%)	103 (91.2%)	43 (78.2%)	
Yes	22 (13.1%)	10 (8.85%)	12 (21.8%)	
Jaundice				<0.001
No	152 (90.5%)	109 (96.5%)	43 (78.2%)	
Yes	16 (9.52%)	4 (3.54%)	12 (21.8%)	
Diabetes				0.556
No	140 (83.3%)	96 (85.0%)	44 (80.0%)	
Yes	28 (16.7%)	17 (15.0%)	11 (20.0%)	
MPD Diameter	4.00 [2.00;8.00]	3.00 [2.00;6.00]	8.00 [4.00;12.0]	<0.001
MPD Diameter				<0.001
<5 mm	94 (56.0%)	76 (67.3%)	18 (32.7%)	
5–9 mm	39 (23.2%)	25 (22.1%)	14 (25.5%)	
≥10 mm	35 (20.8%)	12 (10.6%)	23 (41.8%)	
Mural nodule size	0.00 [0.00;3.25]	0.00 [0.00;0.00]	5.00 [0.00;7.00]	<0.001
Mural nodule size				<0.001
**Without**	113 (67.3%)	95 (84.1%)	18 (32.7%)	
<5 mm	16 (9.52%)	10 (8.85%)	6 (10.9%)	
≥5 mm	39 (23.2%)	8 (7.08%)	31 (56.4%)	
Cyst size	29.0 [20.8;40.0]	27.0 [21.0;37.0]	36.0 [20.0;51.5]	0.050
Cyst size≥30 mm				0.229
No	86 (51.2%)	62 (54.9%)	24 (43.6%)	
Yes	82 (48.8%)	51 (45.1%)	31 (56.4%)	
Cyst size≥40 mm				<0.001
No	125 (74.4%)	96 (85.0%)	29 (52.7%)	
Yes	43 (25.6%)	17 (15.0%)	26 (47.3%)	
Thickened enhanced cyst wall				0.015
No	100 (59.5%)	75 (66.4%)	25 (45.5%)	
Yes	68 (40.5%)	38 (33.6%)	30 (54.5%)	
Abrupt change in caliber of MPD with distal pancreatic atrophy				0.764
No	102 (60.7%)	70 (61.9%)	32 (58.2%)	
Yes	66 (39.3%)	43 (38.1%)	23 (41.8%)	
Acute pancreatitis				0.277
No	132 (78.6%)	92 (81.4%)	40 (72.7%)	
Yes	36 (21.4%)	21 (18.6%)	15 (27.3%)	
Lymphadenopathy				0.039
No	153 (91.1%)	107 (94.7%)	46 (83.6%)	
Yes	15 (8.93%)	6 (5.31%)	9 (16.4%)	

Abbreviations: BD, branch duct; DP, distal pancreatectomy; DPPHR, duodenum‐preserving pancreatic head resection; MD, main duct; MP, middle pancreatectomy; MPD, main pancreatic duct; MT, mixed type; NLR, neutrophil‐to‐lymphocyte ratio; PD, pancreatoduodenectomy; PLR, platelet‐lymphocyte ratio; PNI, prognostic nutritional index; TP, total pancreatectomy.

Of the 25 cases of IPMN with invasive carcinoma (presented in the Appendix 1), 11 were staged as T1aN0M0, 6 were staged as T1bN0M0, 3 were staged as T1cN0M0, and 1 case each was staged as T2N0M0, T3N0M0, T1bN1M0, T2N1M0, and T4N2M0.

### 3.2 Univariate analysis and multivariate analysis

Univariate analysis was performed for the variables shown in Table 3, and seven variables that were significantly different between the benign and malignant IPMN groups were identified. CA19‐9 ≥ 37 U/mL, CEA ≥5 ng/mL, PNI ≤45.875, obstructive jaundice, enhancing mural nodule (<5 mm and ≥5 mm), MPD diameter (5–9 mm and ≥ 10 mm), cyst size ≥40 mm, thickened enhanced cyst wall, and lymphadenopathy were frequently associated with malignant IPMN. Further multivariate analysis showed that CA19‐9 ≥ 37 U/mL, PNI ≤45.875, MPD diameter (5–9 mm and ≥ 10 mm), enhancing mural nodule (<5 mm and ≥5 mm), cyst size *t* ≥ 40 mm remained independent predictors for malignant IPMN.

**TABLE 3:** Univariate and multivariate analyses of risk factors for malignant IPMN.

Characteristic		Univariable OR (95% CI)	*p*‐value	Multivariable OR (95% CI)	*p*‐value
Sex	Male vs. Female	1.45 (0.74–2.83)	0.276		
Location	Head/uncinate vs. Body/tail	0.90 (0.17–4.89)	0.904		
	Diffuse vs. Body/tail	1.94 (0.93–4.06)	0.078		
BMI grade	Underweight vs. Normal	1.96 (0.65–5.67)	0.2		
	Overweight vs. Normal	1.87 (0.90–3.87)	0.092		
	Obesity vs. Normal	2.80 (0.49–16.0)	0.2		
CA19‐9 ≥ 37 U/mL	Yes vs. No	5.38 (2.14–13.58)	<0.001	6.36 (1.48–27.32)	0.013
CEA≥5 ng/mL	Yes vs. No	2.87 (1.16–7.15)	0.023	1.46 (0.35–6.07)	0.601
NLR≥2	Yes vs. No	1.17 (0.61–2.26)	0.639		
PLR≥120	Yes vs. No	1.04 (0.54–2.03)	0.899		
PNI≤45.875	Yes vs. No	2.97 (1.52–5.82)	0.002	2.73 (1.01–7.35)	0.048
Jaundice	Yes vs. No	7.60 (2.32–24.88)	<0.001	5.00 (0.91–27.38)	0.064
Diabetes	Yes vs. No	1.41 (0.61–3.26)	0.420		
Mural nodule size	<5 mm vs. without	3.17 (1.02–9.81)	0.046	8.09 (1.84–35.53)	0.006
	≥5 mm vs. without	20.45 (8.10–51.64)	<0.001	14.93 (4.15–53.65)	0.001
MPD Diameter	5–9 mm vs. <5 mm	2.36 (1.03–5.43)	0.043	6.11 (1.81–20.68)	0.004
	≥10 mm vs. <5 mm	8.09 (3.40–19.25)	<0.001	18.39 (4.63–73.11)	0.001
Cyst size≥30 mm	Yes vs. No	1.57 (0.82–3.01)	0.173		
Cyst size ≥40 mm	Yes vs. No	5.06 (2.42–10.60)	<0.001	3.51 (1.14–10.84)	0.029
Thickened enhanced cyst wall	Yes vs. No	2.37 (1.23–4.58)	0.010	1.87 (0.69–5.10)	0.221
Abrupt change in caliber of MPD with distal pancreatic atrophy	Yes vs. No	1.17 (0.61–2.26)	0.639		
Acute pancreatitis	Yes vs. No	1.64 (0.77–3.51)	0.200		
Lymphadenopathy	Yes vs. No	3.49 (1.17–10.37)	0.024	2.43 (0.53–11.18)	0.254

Abbreviations: BMI, Body mass index, Underweight, <18.5, Normal, 18.5 — 24.9, Overweight, 25 — 29.9, Obesity, ≥30; NLR, neutrophil‐to‐lymphocyte ratio; MPD, main pancreatic duct; PLR, platelet‐lymphocyte ratio; PNI, prognostic nutritional index.

### 3.3 Construction and evaluation of the nomogram

**FIGURE 2:**
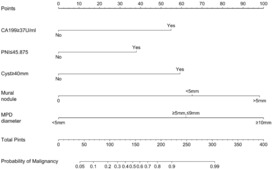
The nomogram for detecting malignant potential in patients with intraductal papillary mucinous neoplasm.

**FIGURE 3:**
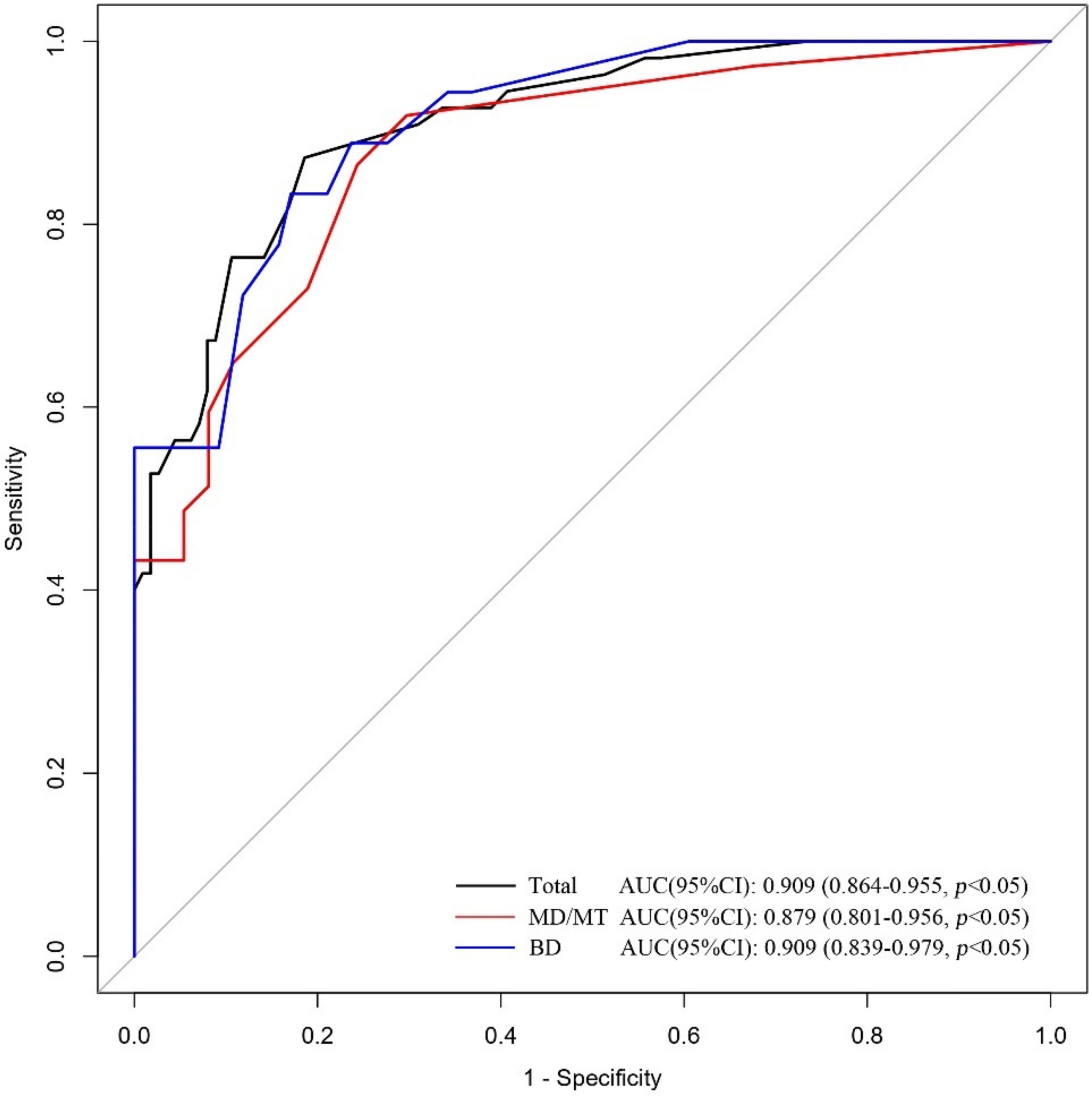
Receiver operating characteristic curve of the nomogram. (AUC refers to area under the curve).

**FIGURE 4:**
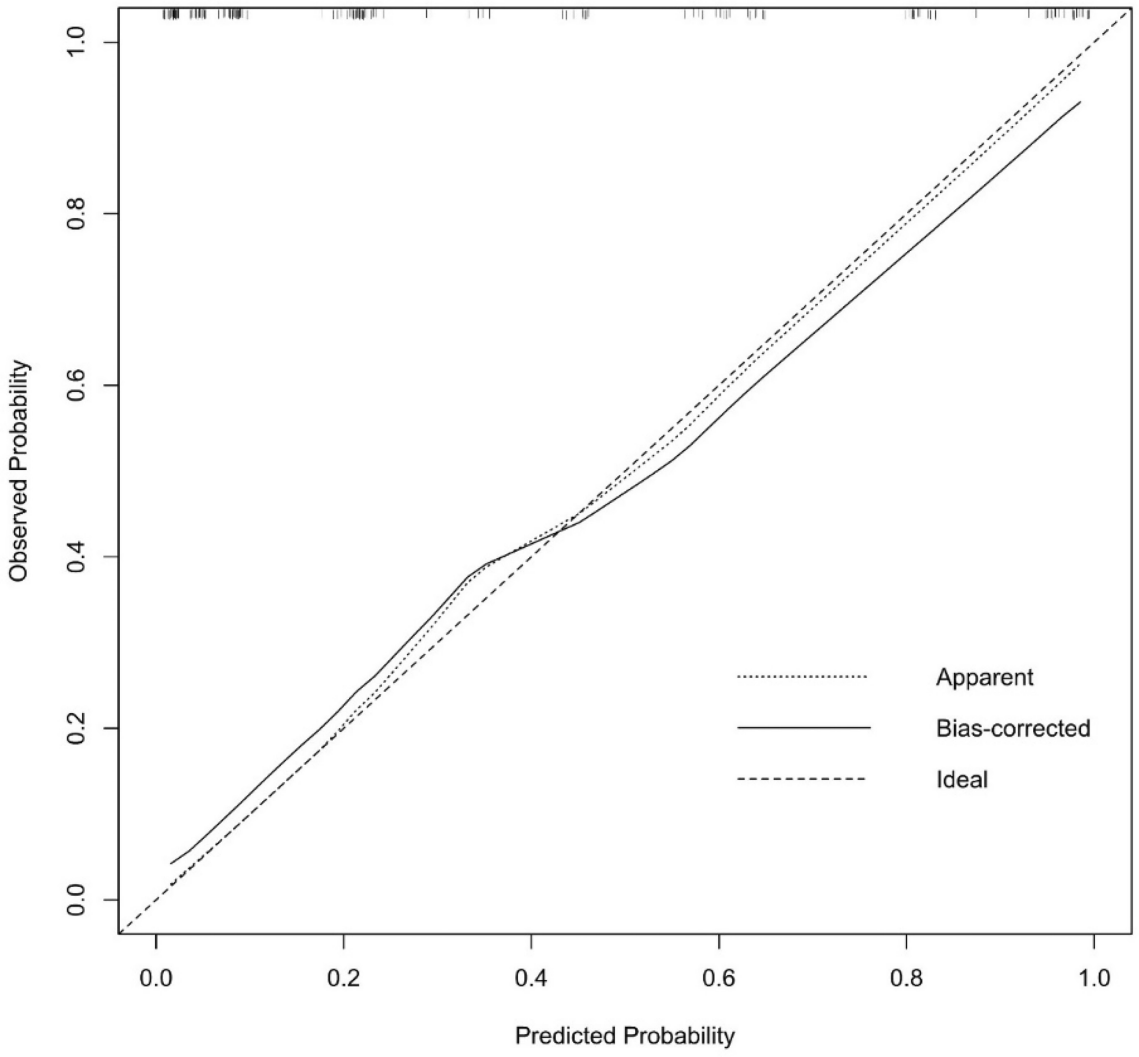
The calibration curve for assessing the accuracy of the nomogram.

**FIGURE 5:**
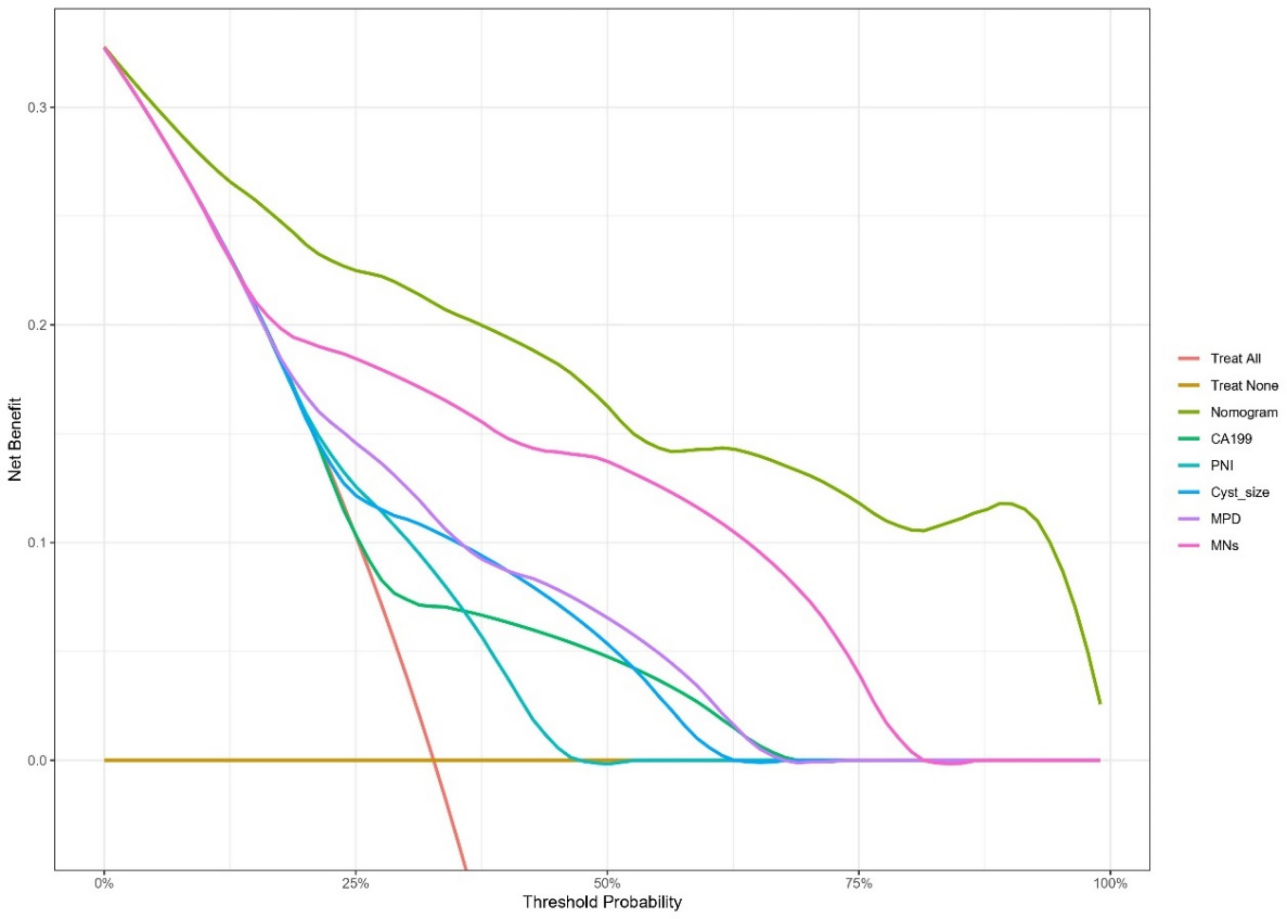
Decision curve analysis (DCA) for the nomogram (The x axis was defined as threshold probability. The threshold probability (Pt) refers to the probability that malignant IPMN will trigger medical intervention, for which the clinician considers the surgical damage acceptable. The y axis represents the net benefit under various threshold probabilities. The brown lines parallel to the x‐axis means all patients receive surveillance, the orange oblique line represents that all patients undergo surgery. The other six different color lines represent different strategies to perform surgery for patients with IPMN, depending on the nomogram or single predictor).

Based on the characteristics that remained statistically significant in the multivariate analysis, a nomogram (Figure 2) was created to predict malignant IPMN in patients prior to surgery. Figure 3 depicted the ROC curve and AUC for the prediction of malignancy in all types (0.909, 95% CI: 0.864–0.955, *p* < 0.05), MD/MT (0.879, 95% CI: 0.801–0.956, *p* < 0.05), and BD type (0.909, 95% CI: 0.839–0.979, *p* < 0.05). The AUC for the prediction of malignancy in all types, MD/MT, and BD type remained 0.875, 0.848, and 0.864, respectively, after internal cross‐validation. Table 4 showed the diagnostic performance of the various probability cut‐off settings. The nomogram achieved the maximum balanced accuracy (84.34%), sensitivity (87.27%), and specificity (81.42%) when the cut‐off was 0.23. The calibration curve was shown in Figure 4, in which the line from the developed nomogram closely resembles the ideal line (diagonal line), indicating favorable predictive accuracy between the actual and predicted probabilities. The decision curve analysis of the predictive nomogram (Figure 5) showed the net benefit of different treatment strategies under various threshold probabilities. When the threshold probability was >0.05, the net benefit of applying the nomogram was always superior to that of the other strategies (treat‐all, treat‐none, and depend on the single predictor).

**TABLE 4:** Diagnostic performance of different cut‐off values of malignant IPMN probability in the predictive nomogram.

Cut‐off	Sen	Spe	PPV	NPV	BA	TP	TN	FN	FP
10%	0.9273	0.6106	0.5368	0.9452	0.7689	51	69	4	44
20%	0.9273	0.6372	0.5543	0.9474	0.7822	51	72	4	41
23%	0.8727	0.8142	0.6957	0.9293	0.8434	48	92	7	21
30%	0.8000	0.8407	0.7097	0.8962	0.8204	44	95	11	18
40%	0.7636	0.8761	0.7500	0.8839	0.8199	42	99	13	14
50%	0.6727	0.9115	0.7872	0.8512	0.7921	37	103	18	10
60%	0.6182	0.9204	0.7907	0.8320	0.7693	34	104	21	9
70%	0.5273	0.9735	0.9062	0.8088	0.7504	29	110	26	3
80%	0.5273	0.9735	0.9062	0.8088	0.7504	29	110	26	3
90%	0.3455	1.0000	1.000	0.7584	0.6727	19	113	36	0

Abbreviations: BA, balanced accuracy, defined as (specificity+ sensitivity)/2; FN, false negative; FP, false positive; NPV, negative predictive value; PPV, positive predictive value; Sen, sensitivity; Spe, specificity; TN, true negative; TP, true positive.

## 4 DISCUSSION

In a multicenter retrospective study,^29^ including 1210 patients diagnosed with IPMN radiologically, Pulvirenti et al. found that between 2000 and 2015, pancreatic cystic lesions, including IPMN detected through CT or MRI, increased annually, and the overall percentage of patients undergoing resection procedures because of radiologically suspicious IPMN appeared to be decreasing. With the updating of relative guidelines, the percentage of identified malignant pathologies decreased, although the percentage of preoperative high‐risk features increased in resected BD‐IPMN, suggesting that these proposed guidelines did not improve our ability to identify malignancy in IPMN. It does not seem to be the perfect choice for clinical practitioners to decide whether to perform surgery or surveillance for patients diagnosed with IPMN radiologically based on guidelines. Therefore, new predictive methods for high‐risk diseases are urgently required to improve patient management. Several previously constructed nomograms have shown excellent predictive power, indicating that dependable nomograms have promising application potential. Shimizu et al. developed a predictive model that included sex, type of IPMN, size of mural nodules (MNs), and pancreatic juice cytology in 2015^17^ and constructed a new model, including three parameters (mural nodule, MPD diameter, and cyst size), based on the 2012 IAP guidelines in 2020,^12^ which performed well in external validation. However, cytological analysis and mural nodule assessment were dependent on invasive examinations (ERCP and EUS, respectively) whose results highly relied on the skills and experiences of the operators. At the same time, the non‐standardized cytology grade used by the model made the nomogram difficult to apply in other centers. Jung et al. developed a predictive model for BD‐IPMN in an international multicenter study in 2016^30^ and further optimized the model in 2019,^14^ which showed excellent discrimination performance in the validation cohort with an AUC of 0.737. Although mural nodule was considered in their model, its size was ignored. Further stratification of MN size might improve the discrimination power of the model. In 2018, Atiyeh et al. developed two separate nomograms for BD‐IPMN and MD‐IPMN based on a multicenter database.^15^ The two models incorporated several parameters, such as age, sex, symptoms, and radiological findings. However, the definition of abdominal symptom seemed too extensive. The 2018 American College of Gastroenterology (ACG) guidelines stated that abdominal symptoms should be carefully attributed to PCNs, including IPMN.^31^ In addition, the model did not consider potential biological markers from laboratory tests. In 2021, Libo et al. constructed a nomogram combining noninvasive radiological imaging and laboratory test features that showed excellent predictive performance for both BD‐IPMN and MD‐IPMN.^28^ However, they did not consider mural nodules, which are one of the most important predictors of malignant IPMN.

We developed a malignancy prediction nomogram combining several variables identified as independent predictors of malignant IPMN in multivariate analysis, of which four were listed as risk factors or surgical indications in the main guidelines.

Notably, the probability of malignancy in MD‐IPMN was clearly higher than that in BD‐IPMN in resected specimens (61.6% vs. 31.1%), according to the 2017 IAP guidelines.^7^ A recent review also highlighted the equivalent risks of various types summarized from a series of previously published studies.^32^ In the present study, 50% (37/74) of the malignant cases were discovered in MD‐IPMN, while only 19.1% were discovered in BD‐IPMN, in agreement with the former reports. IPMN subtypes are mainly determined using preoperative imaging. However, it is the reality that discrepancies between preoperative radiologic criteria and pathologic assessment actually exist in the diagnoses of the subtype of IPMN, which may result in unnecessary resection and missing malignancy in clinical practice.^28^ In some IPMN cases, it is difficult to clearly classify them as MD/MT or BD type.^33^ Therefore, the subtype was not under consideration in the developed model. Although MPD dilatation does not completely indicate MPD involvement, it is the focus that clinicians actually concerned. In the study, dilatation of the MPD was divided into three levels (<5 mm, 5–9 mm, and ≥ 10 mm), and the degree of dilatation was found to be strongly correlated with the risk of malignancy. The MPD dilatation of 5–9 mm showed an odds ratio (OR) of 6.11, while MPD dilatation ≥10 mm showed an OR of 18.39, which was in alignment with series of previous studies. The 2017 IAP guidelines recommend to resect all IPMN with MPD ≥10 mm, and the 2018 ESG guidelines also consider surgical procedure for IPMN with MPD of 5–9 mm. In fact, MPD ≥10 mm showed a PPV of only 65.7%, whereas MPD of 5–9 mm showed a PPV of 35.9% in the present study, which is comparable to the result in Atiyeh's study in 2018.^15^ A retrospective study conducted in 2018 showed that a dilated MPD alone was not associated with an increased incidence of malignancy in patients with IPMN under surveillance.^34^ Thus, it is necessary to consider other risk factors when determining treatment plans for patients with MPD dilatation in IPMN. Eventually, our final nomogram included the stratified MPD diameter, which was assigned a distinct weight according to the degree of MPD dilatation, to better evaluate individual risk by combining other predictors.

Enhancing mural nodules have been proven to be a strong independent risk factor for IPMN in previous studies and are included in the most developed nomogram.^13^, ^15^, ^17^ Distinct from other studies in which mural nodules were assessed using EUS, which was an invasive examination, we detected mural nodules through cross‐sectional imaging (CT/MRI), whose accuracy might be inferior to that of EUS; however, it could be more practical for the widespread application of CT/MRI in most medical institutions. In our study, the presence of an enhancing mural nodule showed a strong correlation with malignant IPMN, and a mural nodule size of 5 mm or more was related to a higher OR than a smaller mural nodule size (14.93 vs. 8.09) when compared to the absence of a mural nodule. As mentioned above, some nomograms only considered the present of MNs, ignoring their sizes, while others neglected low level size of 0–5 mm, although they had considered MNs ≥5 mm. In our nomogram, MNs were stratified into two levels, allowing us to measure the risk of malignancy more precisely, which might have improved the discriminating power of the nomogram.

First proposed by the Japanese scholar, Onodera,^35^ the PNI is closely associated with the clinical outcomes of malignant tumors, including pancreatic ductal adenocarcinoma, as reported in recent studies.^36^, ^37^ In a retrospective study, involving 155 patients with IPMN, Okamura et al. noticed that the PNI of patients with malignant IPMN was significantly lower than that of patients with benign IPMN (42.2 vs. 43.7, *p* = 0.023), and that the PNI value was an independent prognostic predictor of invasive IPMN.^23^ Hata et al. found that PNI ≤45.6 was significantly associated with malignant IPMN in their study.^38^ But whether it is a predictor for the risk of malignant IPMN has not been further investigated. Consistent with previous studies, the PNI value was significantly different between the two subsets of IPMN (benign vs. malignant: 48.5 vs. 42.0, *p* < 0.001). In the present study, we attempted to verify its value in predicting malignancy. To determine the cut‐off values of PNI for predicting malignancy, an ROC curve was employed, which revealed that a cut‐off of 45.875 corresponded to the maximum Youden index, with a sensitivity of 76.1%, specificity of 76.4%, and an AUC of 0.823. Multivariate analysis suggested that PNI ≤ 45.875 was a significant risk factor for malignant IPMN. Previous research has revealed that the preoperative nutritional status is related to the prognosis of pancreatic tumors.^39^ As an important part of the inflammatory response, the immune and nutritional statuses of the system are closely related to the occurrence and development of tumors.^40^ Lymphocytes serve as an important part of the immune system and can eliminate cancer cells and inhibit cancer cell proliferation, invasion, and migration. A reduction in the lymphocyte count may weaken bodily immune functions and reduce the inhibition of cancer cell proliferation and invasion, thus leading to tumor progression, which in turn may inhibit immune function.^41^ The serum albumin level is a simple and effective parameter that reflects nutritional status and is related to immune ability. Inflammatory factors (such as interleukin‐6 and tumor necrosis factor) in the tumor microenvironment can inhibit albumin synthesis in the liver.^42^ A low PNI value may reflect preoperative malnutrition and potential immunocompromised or immune‐suppressed states and is associated with malignant IPMN. However, the underlying mechanisms require further investigation. To the best of our knowledge, this was the first study in which the PNI was verified as an independent risk factor for malignant IPMN and was used to construct a prediction nomogram. Nevertheless, the cut‐off value of the PNI seems to be quite open, and its rationality and reliability deserve further validation by more studies.

Compared with former predictive nomograms, the model we developed combined laboratory examination and noninvasive radiological features that are generally accessible simultaneously, which might enable its widespread adoption and application. In addition, the MPD diameter and mural nodule size were stratified into different levels, which helped to better assess the risk of malignancy and improve the discrimination of the predictive nomogram. It showed excellent capability in predicting malignant IPMN for both BD and MD/MT types. DCA for the predictive nomogram suggests that applying the nomogram to determine the management strategy between aggressive surgery and conservative surveillance according to the risk of malignancy predicted by the model would provide better clinical outcome under most conditions, compared with other strategies depending on the single predictor and two extremes (“treat all patients” and “treat no patient”). Nevertheless, when the threshold probability was lower than 5% or even 10%, the nomogram cannot provide more net benefit. Clinicians should fully consider the patient's individual condition and make decision carefully after multidisciplinary treatment.

However, this study had several shortcomings. First, this was a retrospective study. Only patients who underwent surgery and had pathologically confirmed IPMN were included in the present study. Since patients who were still under monitoring were excluded, the growth rate of the cyst was not investigated. Additionally, due to a lack of pertinent data, we were unable research on some potential predictors, such as the C‐reactive protein‐to‐albumin ratio.^43^ Furthermore, our study was conducted at a single medical center, and the number of patients did not reach a considerable scale. Moreover, external validation of this method was lacking. Prospective studies should be conducted in multiple high‐volume centers to overcome the aforementioned limitations and provide reliable results.

## 5 CONCLUSION

In conclusion, increased serum CA19‐9, low PNI values, cyst size, enhancing mural nodule size, and MPD diameter are independent predictors of malignant disease in IPMN. The nomogram developed in the current study first introduces a new circulation biomarker of PNI and has a favorable performance in predicting malignant IPMN. Employing the model preoperatively might provide suggestions for clinical practitioners to make appropriate decisions for those suffering from IPMN. Since all parameters in the nomogram were gathered from the generally accessible laboratory examination and noninvasive radiological imaging, the model we developed can be applied in most institution. Nevertheless, further prospective studies should be conducted to increase its dependability, and external validation is required to confirm its efficacy.

## ETHICS APPROVAL

The institutional review board of the Tongji Hospital, Tongji Medical College, Huazhong University of Science and Technology approved this study. Written informed consent was waived for the retrospective nature of the study.

## AUTHOR CONTRIBUTIONS

**Xiaorui Huang:** Conceptualization (equal); data curation (equal); formal analysis (equal); investigation (equal); methodology (equal); project administration (equal); resources (equal); visualization (equal); writing – original draft (equal); writing – review and editing (equal). **Tong Guo:** Data curation (equal); methodology (equal); software (equal); supervision (equal). **Zhiwei Zhang:** Conceptualization (equal); project administration (equal); validation (equal). **Ming Cai:** Investigation (equal); validation (equal). **Xinyi Guo:** Data curation (equal). **Jingzhao Zhang:** Investigation (equal). **Yahong Yu:** Project administration (equal); resources (equal); supervision (equal); writing – review and editing (equal).

## FUNDING INFORMATION

This study was supported by grant from the Wuhan applied and basic research project of Wuhan Science and Technology Bureau (2022020801010442).

## CONFLICT OF INTEREST STATEMENT

The authors declare that the research was conducted in the absence of any commercial or financial relationships that could be construed as a potential conflict of interest.

## Data Availability

The data that support the findings of this study are available from the corresponding author upon reasonable request.

## APPENDIX 1 Detailed TNM classification and stage of 25 cases with invasive IPMN.

### 1.1

**Table:**

Case	T	N	M	TNM	Stage
1	1a	0	0	T1aN0M0	I A
2	1a	0	0	T1aN0M0	I A
3	1a	0	0	T1aN0M0	I A
4	1a	0	0	T1aN0M0	I A
5	1a	0	0	T1aN0M0	I A
6	1a	0	0	T1aN0M0	I A
7	1a	0	0	T1aN0M0	I A
8	1a	0	0	T1aN0M0	I A
9	1a	0	0	T1aN0M0	I A
10	1a	0	0	T1aN0M0	I A
11	1a	0	0	T1aN0M0	I A
12	1b	0	0	T1bN0M0	I A
13	1b	0	0	T1bN0M0	I A
14	1b	0	0	T1bN0M0	I A
15	1b	0	0	T1bN0M0	I A
16	1b	0	0	T1bN0M0	I A
17	1b	1	0	T1bN0M0	I A
18	1c	0	0	T1cN0M0	I A
19	1c	0	0	T1cN0M0	I A
20	1c	0	0	T1cN0M0	I A
21	2	0	0	T2N0M0	I B
22	3	0	0	T3N0M0	II A
23	1b	1	0	T1bN1M0	II B
24	2	1	0	T2N1M0	II B
25*	4	2	0	T4N2M0	III

*Note*: IPMN with invasive carcinomas were staged depending on the depth of invasive components, according to the AJCC/TNM classification system (8th edition).
